# Synergistical Performance Modification of Epoxy Resin by Nanofillers and Carboxyl-Terminated Liquid Nitrile–Butadiene Rubber

**DOI:** 10.3390/ma14164601

**Published:** 2021-08-16

**Authors:** Yuanjin Liu, Lixiao Yao, Yue Bu, Qing Sun

**Affiliations:** 1 Air Defense and Anti-Missile College, Air Force Engineering University, Xi’an 710038, China; fishbe@126.com; 2 School of Electrical Engineering, Xi’an University of Technology, Xi’an 710048, China; ylx@xaut.edu.cn (L.Y.); buyue@xaut.edu.cn (Y.B.)

**Keywords:** epoxy resin, liquid rubber, thermally conductive nanofiller, relaxation polarization

## Abstract

Epoxy composite materials are widely used in power equipment. As the voltage level increases, the requirement of material properties, including electrical, thermal, and mechanical, has also increased. Introducing thermally conductive nanofiller to the epoxy/liquid rubber composites system is an effective approach to improve heat performance, but the effects of thermally conductive nanofillers on relaxation characteristics remain unclarified. In this paper, nano-alumina (nano-Al_2_O_3_) and nano-boron nitride (nano-BN) have been employed to modify the epoxy/carboxyl-terminated liquid nitrile–butadiene rubber (epoxy/CTBN) composites system. The thermal conductivity and glass transition temperature of different formula systems have been measured. The effect of the nanofillers on the relaxation behaviors of the resin matrix has been investigated. Results show that the different kinds of nanofillers will introduce different relaxation processes into the matrix and increase the conductivity at the same time. This study can provide a theoretical basis for the synergistic improvement of multiple properties of epoxy resin composites.

## 1. Introduction

The volume of power equipment enlarges with the rising voltage level. At the same time, the requirements for mechanical, thermal, and insulation performances become higher [1,2]. Therefore, insulating materials with synergistically improved multi-performance have become a hotspot [3,4,5]. Epoxy resin has wide application in power equipment, owing to its better molding and impregnation performance. 

As a typical brittle material, enhancing the toughness of epoxy resin is the key to improving the mechanical properties. The main toughening methods are adding liquid rubber or thermoplastic material to the matrix [6,7]. There has been a lot of research on the epoxy/rubber composites system (ERs) including covering morphology, kinetics, electricity, thermodynamic, and mechanics [8,9,10], as well as relaxation behavior of dielectric [11,12,13]. However, the improvement of thermal performance has been limited when solely considering the mentioned toughening method.

Based on the ERs, the addition of thermally conductive fillers can further enhance the thermal performance of the copolymer. Commonly used thermally conductive fillers contain metal, carbonaceous, and ceramic particles [14]. The ceramic particles, such as boron nitride (BN), alumina (Al_2_O_3_), silicon dioxide (SiO_2_), aluminum nitride (AlN), and silicon carbide (SiC), have excellent thermal conductivity but little effect on the electrical properties, indicating a promising application prospect in insulation [15,16]. Better properties of thermal conductivity and breakdown strength were achieved in epoxy composites enhancing by micro-BN and nano-Al_2_O_3_ together. The micro-BN constitutes an internal heat conduction path, while nano-Al_2_O_3_ becomes a bridge connecting BN flaky [17].

Through dual modification by liquid rubber and thermally conductive fillers, a joint improvement of the crack resistance and thermal properties of epoxy resin has been achieved in previous research. Besides, Gong and her co-authors find that the enhancement of the breakdown strength in the ternary copolymer, consisting of epoxy resin, carboxyl-terminated polybutadiene liquid rubber (CTPB), and hBN, is attributed to the accumulation of the spatial charges at the interfacial boundary between the epoxy resin and hBN [4]. Additionally, in epoxy/BN composites toughened by carboxyl-terminated liquid nitrile–butadiene rubber (CTBN), the breakdown strength increases when the content of CTBN ranges from 10% to 15% [5]. However, the effects of thermally conductive fillers on dielectric relaxation of the ERs remain unclarified. The polarization processes reveal the microscopic mechanism of the filler on the electrical properties [18]. Therefore, further study about relaxation polarization is necessary to carry out.

In this research, CTBN is chosen to toughen epoxy resin. Based on the epoxy/CTBN composites system, nano-Al_2_O_3_ and nano-BN are introduced to perform the dual modification, respectively. The thermal conductivity, glass transition temperature, and dielectric spectrum are measured. Besides, the relaxation polarization processes are obtained through peak splitting. By comparing these properties of epoxy resin modified by CTBN and nanofillers, the effect and mechanism of two kinds of thermally conductive nanofillers on cross-linking degree, relative permittivity, and dielectric loss of epoxy/CTBN composites system are analyzed. The research results can provide a theoretical basis for the synergistic improvement of multiple properties of epoxy resin.

## 2. Materials and Sample Preparation

### 2.1. Materials

The epoxy resin used in this study is diglycidyl ether of bisphenol A (DGEBP A) with the epoxy value of 4.4 mmol/g produced by Nantong Xingchen Synthetic Material Co., Ltd., Nantong, China. The curing agent is methyl-hexahydro phthalic anhydride (MeHHPA) produced by Puyang Huicheng Electronic Materials Co., Ltd., Puyang, China. The accelerator is 2-ethyl-4-methylimidazole (2,4-EMI) produced by Shanghai Chemical Industry Development Co., Ltd., Shanghai, China. The carboxyl content of CTBN is 0.61 mmol/g, and the acrylonitrile content is 8.4%, supplied from Qilong Chemical Co., Ltd., Zibo, China. Hexagonal boron nitride (h-BN, 50 nm) was purchased from Dekedaojin Technology Co., Ltd., Beijing, China. The particle size of the alumina is 200 nm produced by Huiguang Metal Material Co., Ltd., Guangzhou, China. All reagents are used without special treatment.

### 2.2. Sample Preparation

The sample preparation of Epoxy/CTBN, Epoxy/CTBN/Al_2_O_3_, and Epoxy/CTBN/BN is as follows. Firstly, epoxy resin and CTBN are mixed in proportion and pre-crosslinked at 150 °C for 1 h. CTBN content is 10 phr (per 100 g of epoxy resin) of epoxy resin. After being pre-crosslinked, the nano-Al_2_O_3_ and nano-BN are introduced respectively with an amount of 3 phr of epoxy resin, and the mixture is put into an ultrasonic mixer at 60 °C for 1 h. Then, curing agent and accelerator are added with the proportion of 73.5 phr and 0.5 phr of epoxy resin, respectively. Vacuum degassing is carried out after mixing. The mixture is then injected into the mold and put into the oven (Shanghai Keheng Industrial Development Co., Ltd., Shanghai, China), and curing is carried out under in sequence at 60 °C /1 h, 80 °C /2 h, 120 °C /4 h, and 160 °C /4 h. After that, the sample is stepwise cooled before being removed from the oven.

### 2.3. Performance Measurement

The samples are immersed in liquid nitrogen for 2 min and then broken by a clamp into smaller pieces. Those sample pieces are coated with gold by ion sputtering for scanning electron microscope (SEM) to observe their microstructure. The distribution of liquid rubber and nanofillers in the resin matrix and the development of cracks could be observed. The SEM used in this manuscript is Merlin Compact (Zeiss Germany, Oberkochen, Germany), in which the electron gun voltage is set to 20 kV.

METTLER DSC822e is used for differential scanning calorimetry (DSC) (Mettler Toledo, Zurich, Switzerland). The measurement temperature is 30 °C to 200 °C with a heating rate of 10 °C/min. NETZSCH LFA447 is used to measure the thermal conductivity of the samples. The sample size is *Φ*12.7 mm with a thickness of 1 mm. The measurement temperature is 25 °C to 225 °C with an interval of 25 °C.

The German Novocontrol company’s concept80 broadband dielectric spectroscopy tester is used to measure the dielectric properties. The sample size is *Φ*40 mm with a thickness of 1 mm. Both surfaces of the samples are coated with gold by the ion sputtering with one side entirely coated, but the other within a circle region of *Φ*30 mm. The frequency range is 10^−1^ to 10^6^ Hz, and the test temperature is 25 °C.

## 3. Results and Discussion

### 3.1. Microstructure and Microcrack Development

**Figure 1:**
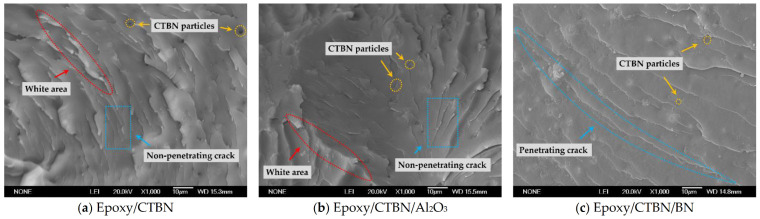
SEM photographs of three kinds of composites system. (**a**) Epoxy/CTBN; (**b**) Epoxy/CTBN/Al_2_O_3_; (**c**) Epoxy/CTBN/BN.

The two-phase structure form by liquid rubber in the resin matrix has a multi-faceted impact on the properties of the composites [19]. Under the synergistic modification of thermally conductive nanofillers, the existence of the nanofillers and their interaction with the rubber particles will further change the properties of composite materials. The SEM cross-sectional photos of the three types of samples with a magnification of 1000 are shown in Figure 1.

Without nanofillers, the section is composed of multiple non-penetrating cracks. The white area in Figure 1a indicates that plastic deformation has occurred during fracture [20]. The liquid rubber is introduced to epoxy resin to form spherical-shaped rubber particles in the cured samples, which hinders the cracks and enhances the toughness of the material. For the sample with nano-Al_2_O_3_, the particles are uniformly dispersed in the resin matrix, and the microscopic morphology of the section is consistent with the epoxy/CTBN composites system. As a contrast, the length of the cracks in the samples with nano-BN increased significantly, and part of the cracks penetrated the entire section, manifesting a brittle fracture. From Figure 1c, the introduction of nano-BN reduces the precipitation of rubber particles and weakens the toughening effect of the liquid rubber.

### 3.2. Thermal Properties Analysis

#### 3.2.1. Glass Transition Temperature

**Figure 2:**
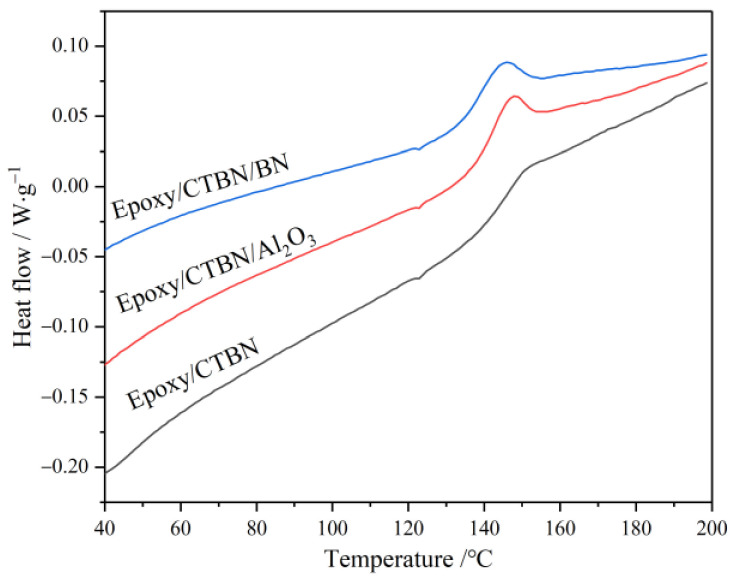
Heat flow curves of three kinds of samples obtained from DSC.

The glass transition temperature (*T*_g_) affects the application range of the material. The *T*_g_ is determined by the heat flow curves obtained from DSC. As illustrated in Figure 2, the baselines shift up nearby 140 °C in all heat flow curves, which is attributed to the remarkable increment of the specific heat capacity after glass transition [21]. In this paper, the inflection point method is utilized to extract the *T*_g_ of different samples, and the results are shown in Table 1. 

The *T*_g_ will decrease slightly in the ERs owing to the diluting effect of liquid rubber on the resin matrix [22]. After the addition of nano-Al_2_O_3_ and nano-BN, the *T*_g_ is further reduced, but the degradation is within 10 °C, which does not affect the application of epoxy composites material. The formation of rubber particles in the resin matrix will reduce the cross-linking degree. With the introduction of nanofillers, the cross-linking degree of the resin matrix has been further reduced, so that the *T*_g_ tends to be lower in Table 1. Besides, the morphology of nano-BN is flaky, and its influence on the cross-linking degree is bigger than that of the granular nano-Al_2_O_3_. Thus, the *T*_g_ of the sample with nano-BN shows a greater drop. Therefore, it can be seen that the morphology of the thermally conductive nanofillers has a greater impact on the *T*_g_ of the composite material.

#### 3.2.2. Thermal Conductivity

**Figure 3:**
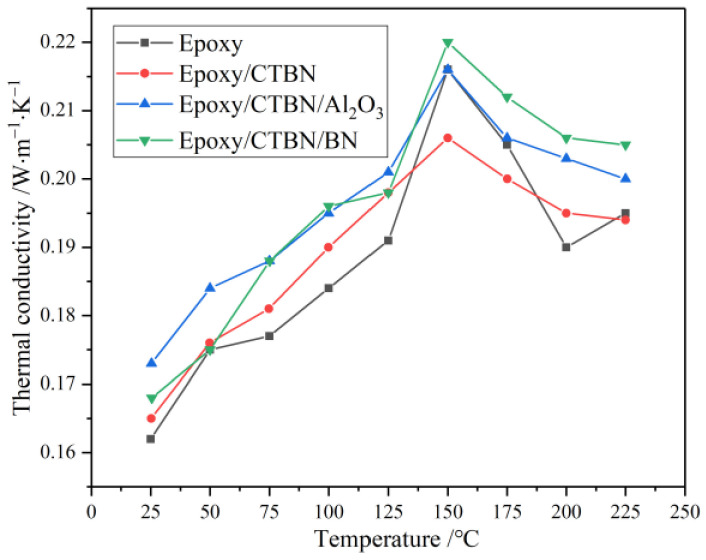
Temperature dependence of thermal conductivity of four kinds of samples.

The thermal conductivity of insulating materials directly affects the temperature and thermal stability of the power equipment. The temperature dependence of thermal conductivity of the four kinds of samples is illustrated in Figure 3.

The maximum thermal conductivity appears near the *T*_g_ in all samples. The thermal conductivity increases with the increase of temperature before the *T*_g_, while exhibiting the opposite trend after the *T*_g_. Except for the different trends near the *T*_g_, the addition of fillers makes the overall thermal conductivity increase. Besides, nano-BN has a more significant increase in thermal conductivity than nano-Al_2_O_3_, which is mainly caused by the difference in the structure of the two fillers. As a high thermal conductivity material, the flaky structure of nano-BN makes it easier to form a thermally conductive network in the resin matrix. Moreover, it has a bigger effect on thermal conductivity when the content is low.

### 3.3. Dielectric Properties Analysis

#### 3.3.1. AC Conductivity

**Figure 4:**
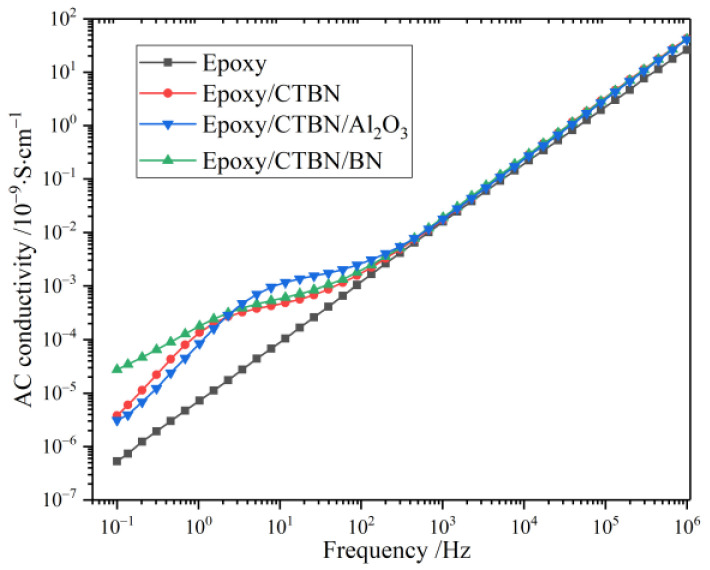
Frequency spectrum of AC conductivity of four kinds of samples at 25 °C.

The AC conductivity reflects the insulating properties under alternating electric fields. According to the low-frequency region in Figure 4, different from the modified samples, there are no obvious fluctuations in the pure epoxy resin. This is mainly because the polarization of the pure epoxy resin is not drastic in the measurement range. For the other three modified samples, the AC conductivity is consistent in the high-frequency region. When behind 1000 Hz, the difference becomes apparent, and the most dramatic change appears in the sample modified by nano-Al_2_O_3_. However, the variation of the AC conductivity in the sample with nano-BN reaches the maximum when in the low-frequency region. In the intermediate frequency region, the rapid variation of the AC conductivity of nano-Al_2_O_3_ samples may be induced by the polarization process, which shows up in a higher frequency. The curves in the low-frequency region indicate that the direct current part of the conductivity in epoxy/CTBN/BN is higher than the two others at room temperature.

#### 3.3.2. Relative Permittivity and Dielectric Loss

**Figure 5:**
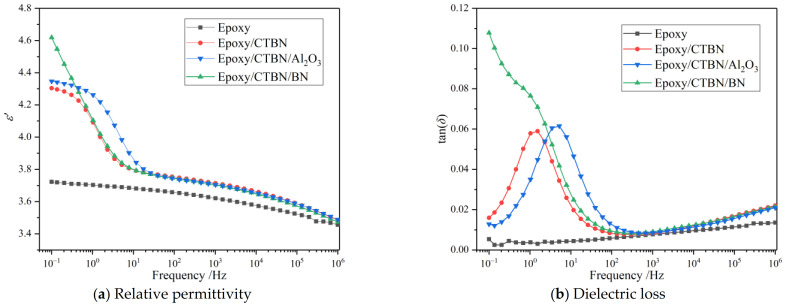
Frequency spectrum of relative permittivity and dielectric loss of four kinds of samples at 25 °C. (**a**) Relative permittivity (*ε*′); (**b**) Dielectric loss (tan*δ*).

The relative permittivity (*ε*′) and dielectric loss (tan*δ*) are two important paraments of the dielectric performance of the insulating material, which determine the potential distribution and heating loss. The frequency spectrum of *ε*′ and tan*δ* of the samples are plotted in Figure 5.

A declining trend of *ε*′ with the increase of frequency in all samples could be observed. The curve of pure epoxy resin has a gentler decline and is significantly lower than the modified samples. The other curves overlap when the frequency is greater than 100 Hz. Compared to the unmodified sample, the distinct decrease step that occurs at the low-frequency region of other curves represents a polarization process. According to the curve of *ε*′, the relaxation strength of the aforementioned polarization in the sample modified by nano-Al_2_O_3_ is greater than the sample only with CTBN. The distinct decrease step that occurs in the low-frequency region is caused by both conduction loss and polarization loss. As indicated in Figure 4, the highest AC conductivity of the Epoxy/CTBN/BN results in the dramatic decline of the ε′ of the sample modified by nano-BN. A two-phase structure will form in the resin matrix after the addition of CTBN, which causes interfacial polarization in the low-frequency region [12], and the introduction of nanofillers changes the relaxation process. 

From the curve of tan*δ* in Figure 5b, the shape and location of the relaxation peak of the interfacial polarization could be confirmed. Compared to the pure epoxy resin, the newly introduced polarization process increases the dielectric loss of the modified samples. In the low-frequency region, an obvious relaxation peak shows in the sample without nanofillers and the sample enhanced by nano-Al_2_O_3_. In contrast, the tan*δ* of the sample modified by nano-BN decreases with the increase of frequency. The peak value of tan*δ* will result in a sharp increase in temperature, which should be avoided during formula design. The tan*δ* curves of the modified samples are consistent during the high-frequency region and are greater than the pure epoxy resin. The introduction of the fillers may increase the branched, side chain, and micro-reactive functional groups in the matrix, which contribute to the increased loss [23].

#### 3.3.3. Dielectric Relaxation

The influence of nanofillers on the *ε*′ and tan*δ* is induced by the microscopic relaxation processes. Therefore, the Havriliak–Negami equation (HN-equation) was employed to analyze the relaxation behaviors of the samples, and the typical form of HN-equation is as follows [23]:

(1) $$\varepsilon^{*}{}_{\mathrm{HN}}\left(\omega \right)=-i\left(\frac{\sigma_{\mathrm{dc}}}{\varepsilon_{0}\omega }\right)+\varepsilon_{\infty }+\sum_{k=1}^{n}\frac{\Delta \varepsilon_{k}}{{\left(1+{\left(i\omega \tau_{k}\right)}^{\beta_{k}}\right)}^{\gamma_{k}}}$$

where ε^*^_HN_(*ω*) is the complex permittivity, *ε*_0_ is the permittivity of the vacuum, *ω* is the angular frequency, *k* represents the number of relaxations behaviors, ∆*ε_k_* is the relaxation strength, *τ_k_* is the relaxation time, *β_k_* and *γ_k_* describe the symmetric and asymmetric broadening of the complex dielectric function, *σ*_dc_ is the DC-conductivity, and *ε_∞_* is the permittivity when *f*→+∞.

**Figure 6:**
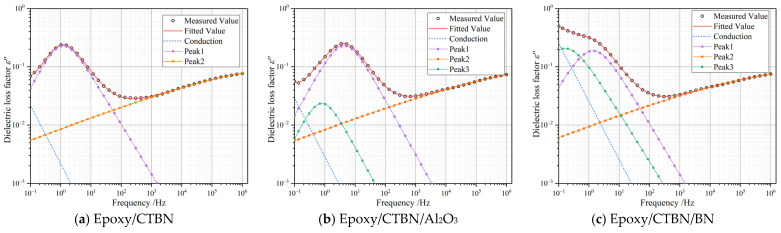
Peak-splitting results of the HN-equation of three kinds of samples at 25 °C. (**a**) Epoxy/CTBN; (**b**) Epoxy/CTBN/Al_2_O_3_; (**c**) Epoxy/CTBN/BN.

The differencing algorithm combined with the least square fitting is used to perform nonlinear fitting on HN-equation [24], and the result obtained is shown in Figure 6. Peak1 represents the interfacial polarization induced by CTBN. With the introduction of nanofillers, the relaxation strength and relaxation time of Peak1 have been changed. Besides, a new relaxation process (Peak3) emerges. The relaxation parameters fitted by the HN-equation are summarized in Table 2.

According to the relaxation parameters of Peak1, the addition of nanofillers will decrease the relaxation time of the interfacial polarization, and the effect of nano-Al_2_O_3_ nanofiller tends to be more evident. This change could be attributed to the variation of permittivity and conductivity. The nano-Al_2_O_3_ nanoparticles could act as the nucleation center of the rubber phase, which could decrease the relaxation time of the interfacial polarization. Instead, the flaky morphology of nano-BN may weaken the nucleation effect of CTBN to a certain extent, resulting in a slight decrease in the strength of Peak1.

After being modified by thermally conductive nanofillers, Peak3 was introduced into the low-frequency region. The relaxation strength of the sample with nano-BN exhibits higher under the same filler contents, while the relaxation time of Peak3 is lower in the sample with nano-Al_2_O_3_. Due to the greater surface area of the BN film, the interface between the BN and resin matrix is larger than the sample modified by nano-Al_2_O_3_, which intensifies the relaxation strength of the Peak3. Furthermore, the DC conductivity increase in the dual-modified samples. Compared with the other two samples, the flaky conductive network is easier to form in the sample with nano-BN. Therefore, the DC conductivity of epoxy/CTBN/BN increased by an order of magnitude.

The above analysis shows that the addition of nanofillers will introduce a new relaxation process, in which relaxation time is longer than the interface polarization between the rubber particles and resin matrix. Besides, under the same additive content, the two-dimensional nanomaterials have a higher specific surface area, which leads to a higher intensity of the relaxation peak. Meanwhile, the introduction of nanofillers will increase the conductivity of the material, and the two-dimensional material is easier to construct a conductive network, resulting in a greater increase of conductivity.

## 4. Conclusions

In this paper, nano-Al_2_O_3_ and nano-BN, two kinds of thermally conductive materials with different dimensions, are employed to enhance the epoxy/CTBN composites system. The thermal performance of the dual-modified composites system has been analyzed, and the dielectric properties have been discussed. The conclusion is as follows:

(1) The addition of nano-Al_2_O_3_ and nano-BN both increases the thermal conductivity. Compared to the granular nano-Al_2_O_3_, the flaky morphology of nano-BN facilitates the formation of the thermal network, resulting in a higher increase of the thermal conductivity after the glass transition temperature. Besides, the glass transition temperature of the sample enhanced by nano-BN declines the most, which indicates that the two-dimensional nanofiller has a greater impact on the cross-linking degree of the resin matrix. Based on this formula system, further research can be applied to basin insulators used in Gas-insulator Metal-enclosed Switchgear (GIS).

(2) A new relaxation polarization process, located in a lower frequency region than interfacial polarization, has been induced by thermally conductive nanofillers. Besides, the AC conductivity rises in the dual-modified composites. According to the larger surface area of the BN-nanofiller, the new relaxation peak tends to have higher relaxation strength, and the increase of the conduction is also the largest.

## Figures and Tables

**Table 1:** Glass transition temperature of four kinds of samples.

Sample Type	Epoxy	Epoxy/CTBN	Epoxy/CTBN/Al_2_O_3_	Epoxy/CTBN/BN
*T*_g_ /°C	152.00 [12]	146.74	142.81	138.84

**Table 2:** Relaxation parameters fitted by the HN-equation of three kinds of samples at 25 °C.

Relaxation Parameters		Epoxy/CTBN	Epoxy/CTBN/Al_2_O_3_	Epoxy/CTBN/BN
Relaxation strength	Peak1	0.505	0.508	0.461
	Peak2	1.282	2.803	2.814
	Peak3	None	0.048	0.532
Relaxation time	Peak1	0.146	0.036	0.117
	Peak2	8.94 × 10^−10^	1.67 × 10^−8^	1.43 × 10^−7^
	Peak3	None	0.207	0.968
DC-conductivity		1.21 × 10^−13^ S·m^−1^	1.60 × 10^−13^ S·m^−1^	1.42 × 10^−12^ S·m^−1^
*β*	Peak1	0.974	0.936	0.873
	Peak2	0.199	0.197	0.202
	Peak3	None	1	0.839
*γ*	Peak1	0.877	1	1
	Peak2	1	0.243	0.191
	Peak3	None	0.95	1

## Data Availability

Data sharing is not applicable. No new data were created or analyzed in this study. Data sharing is not applicable to this article.
